# The real-world safety profile of enfortumab vedotin with or without pembrolizumab: insights from a comparative analysis of FAERS

**DOI:** 10.3389/fimmu.2026.1831172

**Published:** 2026-07-24

**Authors:** Heng Chen, Juanjuan Huang, Gefei He

**Affiliations:** Department of Pharmacy, The First Hospital of Changsha (The Affiliated Changsha Hospital of Xiangya School of Medicine, Central South University), Changsha, Hunan, China

**Keywords:** enfortumab vedotin, FAERS, pembrolizumab, pharmacovigilance, urothelial carcinoma

## Abstract

**Introduction:**

The combination of enfortumab vedotin and pembrolizumab (EV+P) has revolutionized advanced urothelial carcinoma treatment, yet their combined real-world safety profile remains insufficiently characterized. This study aimed to quantitatively compare the adverse event (AE) landscapes of EV+P and EV monotherapy using the FAERS data.

**Methods:**

A case/non-case study design was conducted on FAERS data from 2020 Q4 to 2025 Q3. Disproportionality signals were evaluated at the System Organ Class (SOC), High-Level Group Term (HLGT), and Preferred Term (PT) levels. Time-to-onset was analyzed using the Kaplan–Meier method, and narrative review was performed to validate pneumonitis signals.

**Results:**

We identified 3, 004 and 2, 265 AE reports for EV and EV+P, respectively. EV+P demonstrated significantly higher risks across nine SOCs, most notably in endocrine (OR: 5.88), immune system (OR: 2.03), and respiratory (OR: 1.71) disorders. At the PT level, EV+P was strongly associated with hepatitis (OR: 14.79), immune-mediated enterocolitis (OR: 11.77), adrenal insufficiency (OR: 9.14), and pneumonitis (OR: 2.75). Potential novel signals like hearing disorders (OR: 10.64) and dental/gingival conditions (OR: 9.31) were noted but lacked statistical robustness post-correction. Literature validation confirmed a higher incidence of any-grade pneumonitis with EV+P (10% vs. 3.5%). Furthermore, EV+P suggested a potentially earlier onset for several toxicities, including eye (FDR P = 0.047) and skin disorders (FDR P = 0.011).

**Conclusion:**

EV + P demonstrates a broader and more intense AE spectrum than EV monotherapy, driven largely by immune-related toxicities and potential synergistic organ injury. These findings emphasize the need for vigilant clinical monitoring and early intervention when employing this combination therapy.

## Introduction

Urothelial carcinoma (UC) is a major global health burden with high mortality in advanced stages (1, 2). For decades, platinum-based chemotherapy was the standard of care (3). Recently, the therapeutic landscape for metastatic UC (mUC) has been transformed by immune checkpoint inhibitors (ICIs) and antibody-drug conjugates (ADCs). Among these, enfortumab vedotin (EV), an ADC directed against Nectin-4, has demonstrated notable efficacy as monotherapy in patients who progressed after platinum-based chemotherapy and an ICI (4). EV binds to Nectin-4-expressing tumor cells, is internalized, and releases monomethyl auristatin E (MMAE), resulting in microtubule disruption and cytotoxic cell death (5). Common toxicities associated with EV monotherapy include alopecia, peripheral sensory neuropathy, and pruritus (6).

Selecting effective treatments for platinum-ineligible patients with locally advanced or metastatic UC (la/mUC) remains challenging. However, combining the targeted cytotoxicity of EV with the immune-potentiating effects of pembrolizumab (EV+P) has shown remarkable synergistic potential. Clinical trials, including EV-103 and the phase 3 EV-302 trial, demonstrated superior objective response rates, progression-free survival, and overall survival compared to traditional chemotherapy (7, 8). Consequently, EV+P has been established as a new first-line standard for la/mUC. However, this enhanced efficacy is accompanied by a more complex toxicity profile. EV-related AEs, such as rash and neuropathy, occur alongside a wide spectrum of immune-related AEs (irAEs) linked to pembrolizumab, including pneumonitis, endocrinopathies, and hepatitis (9). Notably, the EV+P group exhibited higher rates of dose reductions and treatment discontinuations due to toxicities compared to chemotherapy (7).

Although clinical trials are essential for drug approval, they are conducted in strictly controlled settings with highly selected populations. Therefore, real-world evidence is vital for assessing safety profiles across broader, more heterogeneous patient demographics. The FDA Adverse Event Reporting System (FAERS), a large post-marketing pharmacovigilance database containing millions of spontaneously reported AEs, offers a valuable resource for detecting rare, novel, or unexpectedly frequent AEs.

While previous pharmacovigilance studies have independently evaluated the safety profiles of EV or ICIs like pembrolizumab (10–12), the real-world safety landscape of their concurrent use remains largely unexplored. With EV+P established as the new first-line standard for la/mUC, understanding how this combination alters the overall toxicity profile is clinically urgent. Therefore, this study aimed to address this gap by performing a FAERS-based disproportionality analysis specifically comparing the AE profiles of EV+P versus EV monotherapy. The findings may help refine clinicians’ understanding of treatment-related risks and support safer, evidence-based use of the EV + P combination in clinical practice.

## Methods

### Data source and acquisition

The present retrospective investigation leveraged the FAERS, a premier global pharmacovigilance database containing over 28 million post-marketing safety records. We interrogated this extensive repository to aggregate AE data covering the period from the fourth quarter of 2020 through the third quarter of 2025. The study dataset was constructed by linking seven distinct ASCII files via “PRIMARYID” and “CASEID” identifiers: patient demographics (DEMO), pharmacological information (DRUG), adverse reactions (REAC), clinical outcomes (OUTC), report origins (RPSR), therapy duration (THER), and drug indications (INDI). Reaction terms were mapped to Preferred Term (PTs), and PTs were further aggregated to their corresponding High-Level Group Term (HLGT) and System Organ Classe (SOC) according to the standard Medical Dictionary for Regulatory Activities (MedDRA) (version 27) hierarchy. Institutional Review Board approval was waived as the research relied exclusively on de-identified, public-domain data.

### Data pre-processing and extraction

**Figure 1:**
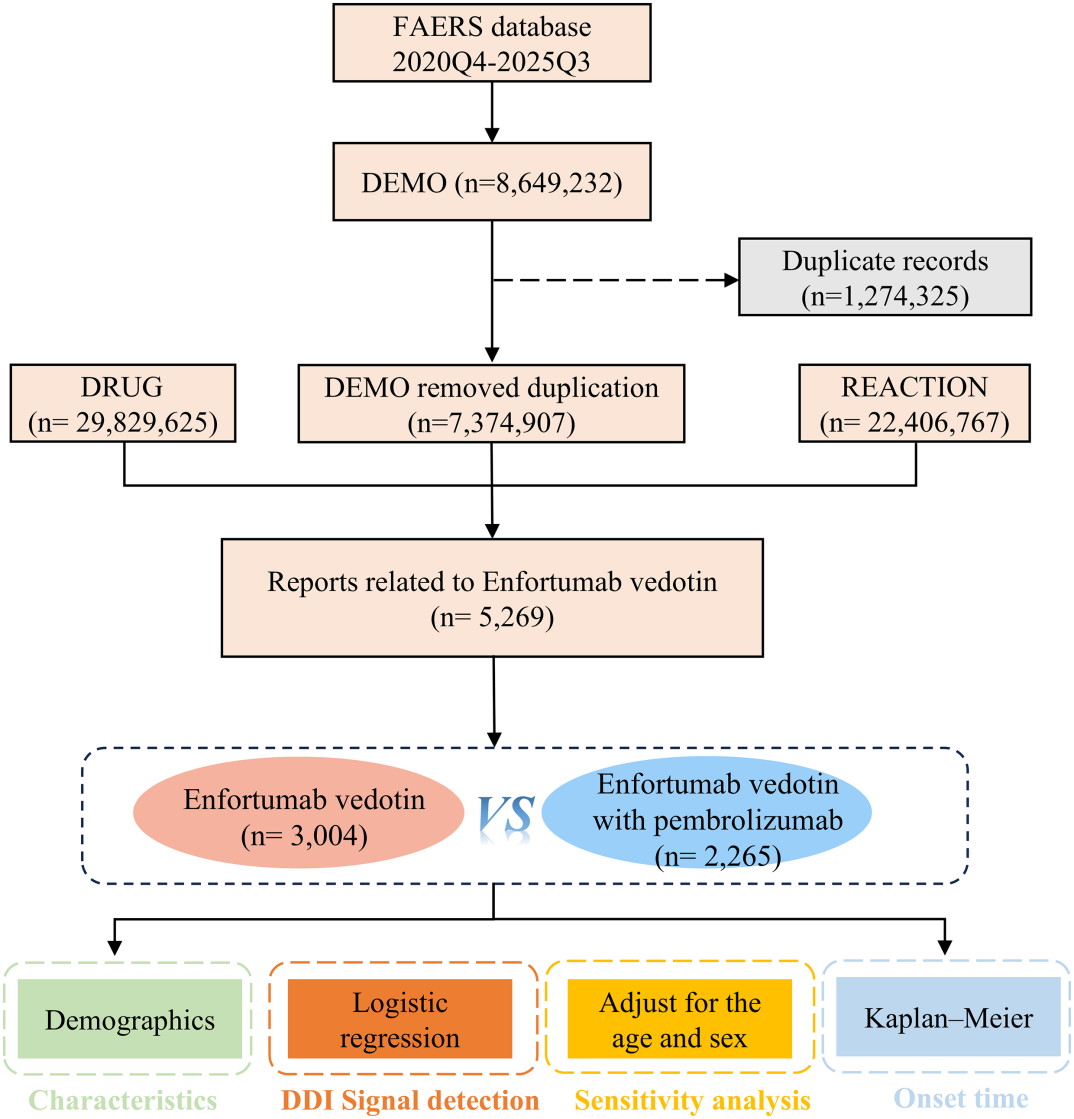
The flow chart of the study.

Raw data files were downloaded from the official FDA portal (https://fis.fda.gov/extensions/FPD-QDE-FAERS/FPD-QDE-FAERS.html) and imported into a MySQL 8.0, yielding an initial dataset of 8, 649, 232 reports. To maintain analytical rigor, a deduplication protocol aligned with FDA recommendations was executed (13). In instances of multiple entries sharing the same CASEID, priority was given to the record with the most recent FDA received date (FDA_DT) and the highest PRIMARYID. This cleansing process resulted in the elimination of 1, 274, 325 redundant records (**Figure 1**). Target cases were identified through a dual-keyword search strategy encompassing the trade name and the generic name. For example, enfortumab vedotin reports were retrieved using the following query logic: DRUGNAME LIKE ‘%enfortumab%’ OR PROD_AI LIKE ‘%enfortumab%’ OR DRUGNAME LIKE ‘%Padcev%’ OR PROD_AI LIKE ‘%Padcev%’. EV+P exposure was operationally defined by the concurrent presence of both enfortumab vedotin and pembrolizumab recorded as suspect drugs within the same FAERS report. To evaluate the influence of drug role coding on the robustness of the results, we performed a sensitivity analysis restricted to Primary Suspect reports. In this analysis, reports were retained only when the target exposure-defining drug was coded as Primary Suspect.

### Narrative review to validate risk signals

To validate the identified risk signals, a comprehensive PubMed search was conducted to identify clinical trials published in the past five years evaluating EV monotherapy or EV combined with pembrolizumab. The search incorporated both MeSh terms and free-text keywords. Abstracts from recent ESMO and ASCO meetings were also reviewed for unpublished data. Eligible studies reported pneumonitis, immune-mediated lung disease, interstitial lung disease, or organizing pneumonia related to EV or EV + P. Data extracted included study name, cancer type, treatment arm, sample size, and pneumonitis incidence (any grade and grade ≥ 3). For overlapping cohorts, the study with the larger sample or latest publication was retained. The incidence was then derived as the ratio of patients with pneumonitis to the total number of patients in each respective treatment group.

### Statistical analysis

Baseline characteristics of the study population were calculated. For categorical data, we reported counts alongside proportions, whereas continuous data were represented by median values and interquartile ranges (IQRs). To assess the safety profile of EV with or without pembrolizumab, we used a case/non-case study design. This evaluation was carried out across multiple hierarchy levels, specifically PT, HLGT, and SOC, with the aim of comparing the reporting frequency of specific drug-event pairs against a control group. Logistic regression was then used to compare the odds ratio (OR) of each AE in the EV plus pembrolizumab group with those in the EV monotherapy group. EV monotherapy served as the reference group. Therefore, an OR > 1 indicated that the corresponding AEs were more frequently reported in the combination therapy group than in the monotherapy group. To account for the increased risk of Type I errors arising from multiple comparisons across numerous MedDRA terms, *P* values were adjusted using the Benjamini-Hochberg False Discovery Rate (FDR) method. A significant disproportionality signal was defined when the lower limit of the 95% confidence interval (CI) for OR (OR_025_) exceeded 1 and the FDR adjusted *P* value was < 0.05. To minimize the impact of potential confounders, we performed multivariable logistic regression to calculate adjusted ORs and their 95% CIs. Age and sex were included as covariates in the model to adjust for baseline demographic imbalances. To further assess whether sparse reporting could inflate disproportionality signals, we performed an additional shrinkage sensitivity analysis.

Furthermore, the time to onset for an AE was calculated as the duration between the initiation of therapy (START_DT) and the AE occurrence date (EVENT_DT). Due to the inherent missing or inaccurate date fields in the FAERS database, a complete-case analysis was conducted for the time-to-onset evaluation. Cumulative incidence curves for AEs were generated using the Kaplan–Meier approach, where the x-axis denotes time since treatment initiation and the y-axis indicates the cumulative probability of AE occurrence. To assess the statistical significance of differences in AE incidence profiles between two groups, log-rank P values were adjusted using the FDR method and reported as FDR P values. A significance threshold of P < 0.05 was adopted.

Two researchers independently managed data extraction via MySQL 8.0 and executed all statistical computations using Python 3.10 and SPSS 27.0.

## Results

### Demographic information

**Figure 2:**
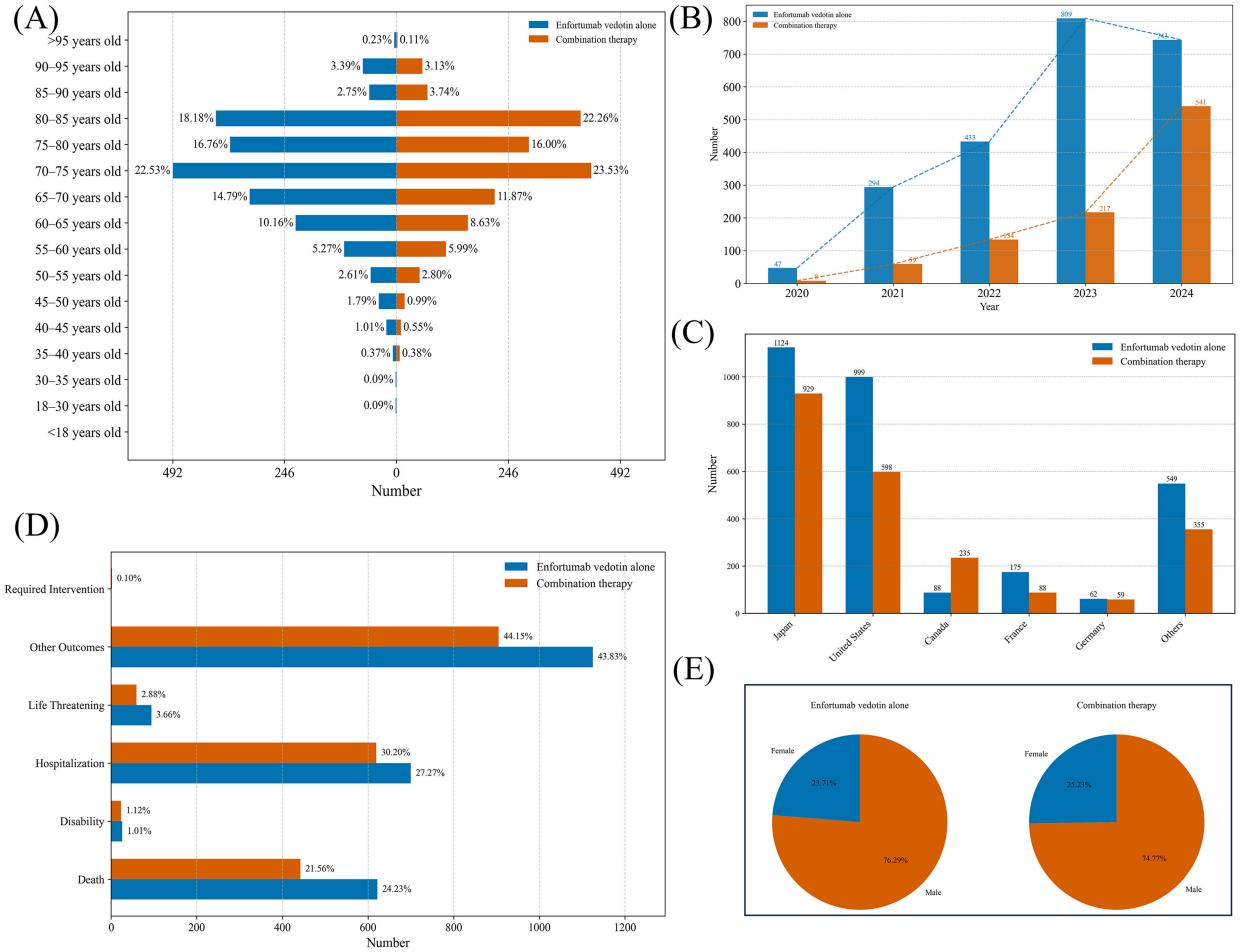
Demographic information of patients treated with enfortumab vedotin with or without pembrolizumab. **(A)** Age distribution of the reported cases; **(B)** Annual distribution of the number of cases; **(C)** Geographic distribution of the reports by country; **(D)** Distribution of the patient outcomes; **(E)** Sex distribution of the patients.

3, 004 cases of AE reports for EV monotherapy and 2, 265 cases for combination therapy were identified from the FAERS database (2020 Q4–2025 Q3) (**Figure 1**). AE reports for combination therapy increased steadily over time, whereas reports for EV monotherapy peaked in 2023 and declined in 2024 (**Figure 2B**). In both groups, patients were primarily aged ≥65 years, with the highest proportion in the 70-75-year subgroup (22.53% and 23.53%, respectively; **Figure 2A**). Males accounted for most reports, with a male-to-female ratio of approximately 3:1 (**Figure 2E**). For EV monotherapy, the highest number of AE reports originated from Japan, the United States, and France, whereas for the combination therapy, the top contributors were Japan, the United States, and Canada (**Figure 2C**). Regardless of the serious outcome, the most common was initial or prolonged hospitalization (EV monotherapy: 27.27%; combination therapy: 30.20%), followed by death (EV monotherapy: 24.23%; combination therapy: 21.56%) (**Figure 2D**).

### Significant risk signals at the SOC level

**Figure 3:**
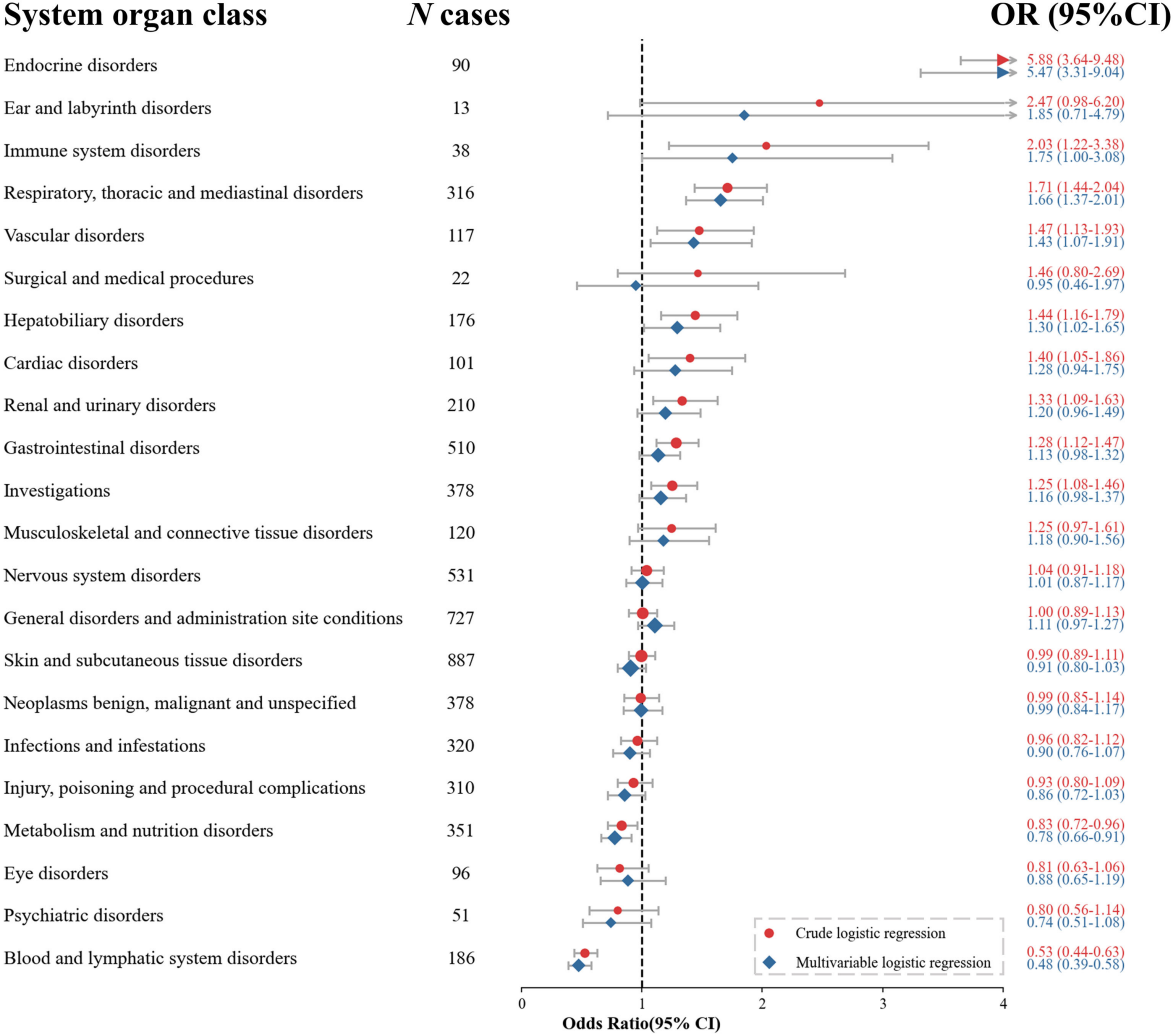
The odds ratio of AEs for combination therapy compared to monotherapy at the SOC level. EV monotherapy was used as the reference group. ORs greater than 1 indicate that the corresponding AEs were more frequently reported in the combination therapy group than in the monotherapy group. Crude ORs were estimated using univariable logistic regression, whereas adjusted ORs were derived from multivariable logistic regression adjusted for age and sex.

**Figure 3** illustrated the SOC profile of AE signals associated with EV monotherapy compared with combination therapy. The results are presented in descending order based on the OR values. Among the 22 SOCs analyzed, 9 demonstrated statistically significant differences (FDR *P* < 0.05). The strongest signal was observed for endocrine disorders (OR: 5.88; 95%CI: 3.64-9.48), followed by immune system disorders (OR: 2.03; 95%CI: 1.22-3.38) and respiratory, thoracic, and mediastinal disorders (OR: 1.71; 95%CI: 1.44-2.04). Additional significant signals were identified in vascular disorders, hepatobiliary disorders, cardiac disorders, renal and urinary disorders, gastrointestinal disorders, and investigations. After adjusting for age and sex using multivariable logistic regression, the sensitivity analysis results were highly consistent with the primary unadjusted analysis (**Figure 3**). The strongest adjusted signal remained endocrine disorders (OR: 5.47; 95% CI: 3.31-9.04). The additional ROR shrinkage analysis confirmed the directional consistency of all nine primary positive signals at the SOC level, with representative findings including endocrine disorders (sROR: 3.95; 95% CI: 2.59-6.02) (**Supplementary Figure 1**).

### Sensitivity analysis restricted to primary suspect reports

To assess whether the inclusion of non-Primary Suspect drug reports influenced the main findings, we performed a sensitivity analysis restricted to reports in which the target exposure-defining drug was coded as Primary Suspect. After this restriction, 4, 187 reports were retained from the original 5, 269 reports, accounting for approximately 79.5% of the primary analytic population. As shown in **Supplementary Figure 2**, the SOC-level results were directionally consistent with the primary analysis. The major organ-system differences observed in the full dataset remained stable after restricting the analysis to Primary Suspect reports, supporting the robustness of the comparative safety findings.

### Significant safety signals at the HLGT level

Further evaluation of AE signals at the HLGT level was conducted, as detailed in **Table 1**. After applying the Benjamini-Hochberg FDR correction, a total of 15 signals remained robustly significant (FDR *P* < 0.05). The most robust signals were identified within the gastrointestinal disorders, encompassing three categories, such as salivary gland conditions (OR: 3.59, 95%CI: 2.21-5.81) and gastrointestinal inflammatory conditions (OR: 2.76, 95%CI: 1.95-3.90). Notably, the signal for endocrine disorders emerged primarily from adrenal gland disorders (OR: 9.21, 95%CI: 3.90-21.73) and thyroid gland disorders (OR: 6.59, 95%CI: 3.21-13.53), with both ranking among the more pronounced risks. And the signal for immune system disorders was supported by immune disorders NEC (OR: 3.56, 95%CI: 1.65-7.68). For respiratory, thoracic and mediastinal disorders, significant risks were associated with respiratory tract signs and symptoms (OR: 2.68, 95%CI: 1.56-4.60) and lower respiratory tract disorders (excl obstruction and infection) (OR: 2.56, 95%CI: 1.99-3.30). In the vascular disorders and cardiac disorders SOCs, high-risk signals included vascular hypertensive disorders (OR: 3.07, 95%CI: 1.46-6.47) and myocardial disorders (OR: 5.55, 95%CI: 2.43-12.70). The strongest risk signal like hearing disorders (OR: 10.64, 95%CI: 1.33-85.16) and dental/gingival conditions (OR: 9.31, 95%CI: 1.14-75.72) were noted but lacked statistical robustness post-correction.

**Table 1:** Evaluation of adverse event signals at the HLGT level.

Adverse event	N	OR (95%CI)	FDR *P*
Hearing disorders	8	10.64 (1.33-85.16)	0.124
Dental and gingival conditions	7	9.31 (1.14-75.72)	0.161
Adrenal gland disorders	41	9.21 (3.90-21.73)	< 0.001
Thyroid gland disorders	44	6.59 (3.21-13.53)	< 0.001
Neuromuscular disorders	13	5.77 (1.64-20.29)	0.045
Myocardial disorders	29	5.55 (2.43-12.70)	< 0.001
Cardiac and vascular investigations (excl enzyme tests)	94	4.29 (2.84-6.50)	< 0.001
Seizures (incl subtypes)	15	4.00 (1.45-11.02)	0.05
Hypothalamus and pituitary gland disorders	12	3.99 (1.29-12.40)	0.088
Salivary gland conditions	61	3.59 (2.21-5.81)	< 0.001
Immune disorders NEC	24	3.56 (1.65-7.68)	0.011
Leukaemias	10	3.33 (1.04-10.62)	0.17
Vascular hypertensive disorders	23	3.07 (1.46-6.47)	0.027
Gastrointestinal inflammatory conditions	99	2.76 (1.95-3.90)	< 0.001
Respiratory tract signs and symptoms	40	2.68 (1.56-4.60)	0.003
Therapeutic and nontherapeutic effects (excl toxicity)	150	2.59 (1.96-3.42)	< 0.001
Lower respiratory tract disorders (excl obstruction and infection)	182	2.56 (1.99-3.30)	< 0.001
Vascular disorders NEC	20	2.42 (1.16-5.07)	0.096
Procedural related injuries and complications NEC	32	2.25 (1.27-3.98)	0.04
Enzyme investigations NEC	24	2.13 (1.12-4.08)	0.108
Renal and urinary tract investigations and urinalyses	31	2.07 (1.18-3.64)	0.075
Exocrine pancreas conditions	26	1.82 (1.01-3.30)	0.18
Hepatic and hepatobiliary disorders	166	1.64 (1.30-2.07)	< 0.001
Muscle disorders	45	1.58 (1.02-2.45)	0.202
Decreased and nonspecific blood pressure disorders and shock	58	1.52 (1.04-2.23)	0.165
Gastrointestinal motility and defaecation conditions	227	1.44 (1.19-1.76)	0.137
Miscellaneous and site unspecified neoplasms malignant and unspecified	309	1.23 (1.04-1.44)	0.002

FDR P: P-value after Benjamini-Hochberg FDR correction for multiple testing.

### Top 30 risk signals at the PT level

At the PT level, the top 30 significant risk signals (ranked by OR) were presented in **Table 2**. Specifically, AEs related to vascular disorders involved the highest number of disproportionality signals, which were mainly associated with blood pressure fluctuations, and the strongest was blood pressure systolic increased (OR: 16.16, 95%CI: 4.97-52.53). Furthermore, the top three strongest risk signals also included hepatitis (OR: 14.79, 95%CI: 4.53-48.28) within the hepatobiliary disorders, along with heart rate decreased (OR: 13.37, 95%CI: 3.12-57.27) from cardiac disorders. The disproportionality signals related to endocrine disorders primarily included adrenal insufficiency (OR: 9.14, 95%CI: 3.57-23.41) and hypothyroidism (OR: 4.69, 95%CI: 2.13-10.30). For immune system disorders, the main PTs involved were immune-mediated enterocolitis (OR: 11.77, 95%CI: 4.18-33.17) and immune-mediated lung disease (OR: 4.80, 95%CI: 1.78-12.96). Positive PTs under respiratory, thoracic and mediastinal disorders were represented by interstitial lung disease (OR: 3.14, 95%CI: 2.16-4.57) and pneumonitis (OR: 2.75, 95%CI: 1.61-4.71), while gastrointestinal disorders manifested as colitis (OR: 4.48, 95%CI: 2.35-8.56), dry mouth (OR: 3.59, 95%CI: 2.21-5.81), among others.

**Table 2:** Top 30 disproportionality signals at the PT level.

Adverse event	N	OR (95%CI)	FDR *P*
Blood pressure systolic increased	36	16.16 (4.97-52.53)	< 0.001
Hepatitis	33	14.79 (4.53-48.28)	< 0.001
Heart rate decreased	20	13.37 (3.12-57.27)	0.004
Blood pressure diastolic decreased	18	12.02 (2.79-51.87)	0.006
Immune-mediated enterocolitis	35	11.77 (4.18-33.17)	< 0.001
Therapy partial responder	59	11.45 (5.22-25.12)	< 0.001
Drug ineffective for unapproved indication	21	9.36 (2.79-31.42)	0.003
Adrenal insufficiency	34	9.14 (3.57-23.41)	< 0.001
Transitional cell carcinoma metastatic	17	7.56 (2.21-25.84)	0.009
Blood pressure fluctuation	23	6.15 (2.34-16.21)	0.002
Blood pressure increased	32	6.14 (2.70-13.93)	< 0.001
Abdominal pain upper	17	5.67 (1.91-16.88)	0.011
Tumour lysis syndrome	17	5.67 (1.91-16.88)	0.011
Myocarditis	20	5.34 (2.00-14.26)	0.006
Oedema	29	4.86 (2.22-10.64)	< 0.001
Immune-mediated lung disease	18	4.80 (1.78-12.96)	0.011
Hypothyroidism	28	4.69 (2.13-10.30)	0.001
Colitis	40	4.48 (2.35-8.56)	< 0.001
Blood pressure decreased	20	3.81 (1.61-9.04)	0.013
Dry mouth	61	3.59 (2.21-5.81)	< 0.001
Ageusia	24	3.21 (1.53-6.72)	0.012
Interstitial lung disease	92	3.14 (2.16-4.57)	< 0.001
Hypertension	23	3.07 (1.46-6.47)	0.016
Pneumonitis	41	2.75 (1.61-4.71)	0.002
Product use issue	66	2.70 (1.77-4.12)	< 0.001
Liver disorder	25	2.22 (1.17-4.23)	0.054
Paraesthesia	25	2.22 (1.17-4.23)	0.054
Oedema peripheral	34	2.16 (1.25-3.74)	0.025
Constipation	59	1.76 (1.19-2.60)	0.023
Diarrhoea	181	1.48 (1.19-1.85)	0.003

FDR P: P-value after Benjamini-Hochberg FDR correction for multiple testing.

### Incidence of treatment-related pneumonitis

**Figure 4:**
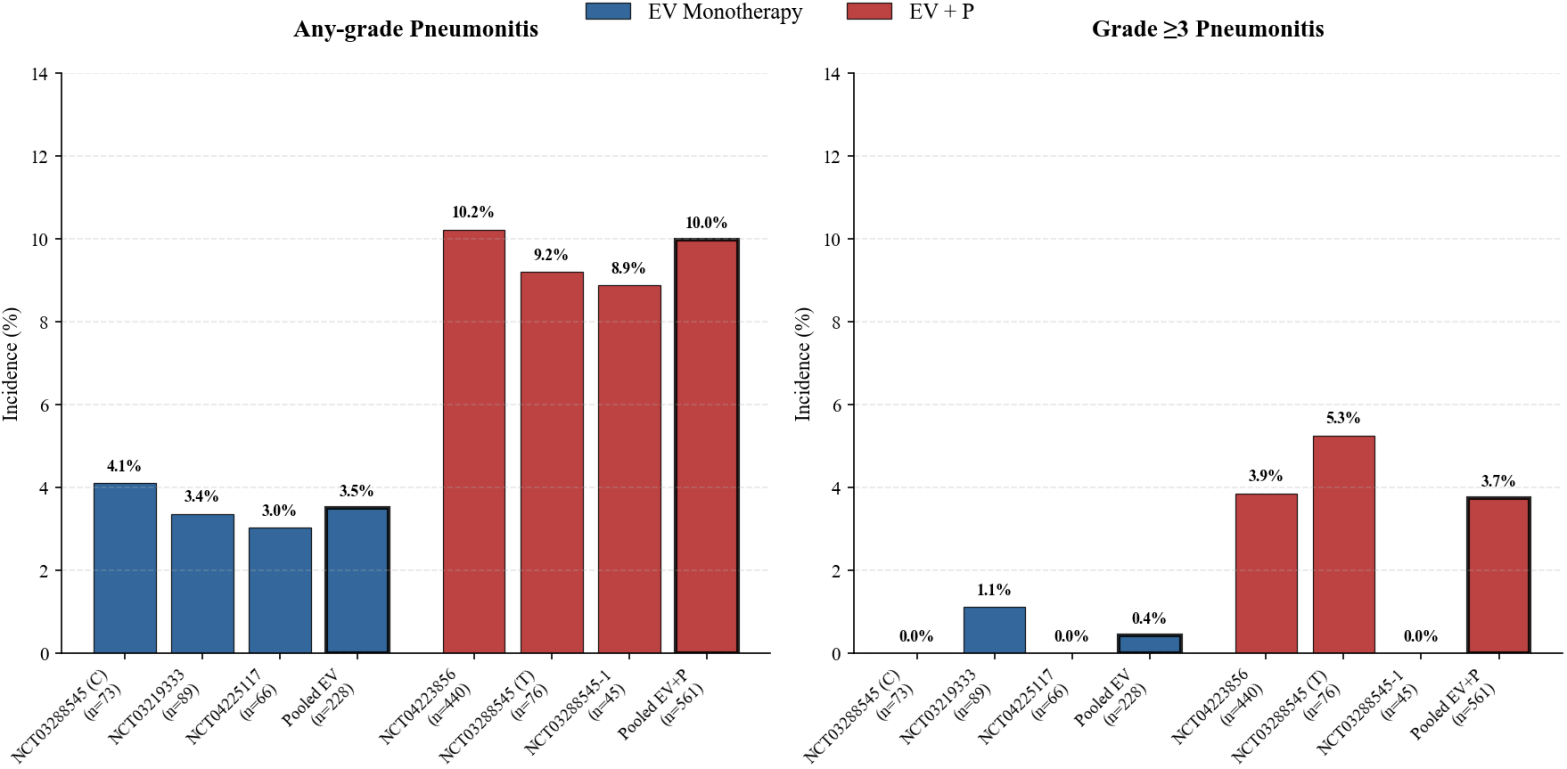
Comparison of pneumonitis risk between enfortumab vedotin and enfortumab vedotin plus pembrolizumab.

A pooled analysis of 5 studies (8, 14–17), encompassing 228 patients receiving EV monotherapy and 561 patients receiving EV + P, was performed to evaluate the incidence of treatment-related pneumonitis (**Figure 4**). The synthesized data demonstrated a significantly higher incidence of any-grade pneumonitis with EV + P (10%) compared to EV monotherapy (3.5%). Similarly, the incidence of grade ≥3 pneumonitis was markedly elevated in the combination therapy group (3.7% for EV + P vs. 0.4% for EV monotherapy).

To further differentiate pneumonitis from infectious or malignant pulmonary causes, a focused PT-level regression analysis of pulmonary-related events was performed via FAERS. Positive signals were concentrated in pneumonitis, interstitial lung disease, and immune-mediated lung disease, whereas pneumonia, pneumonia aspiration, metastases to lung, and lung disorder did not show significant positive signals (**Supplementary Table 1**).

### The time to onset

**Figure 5:**
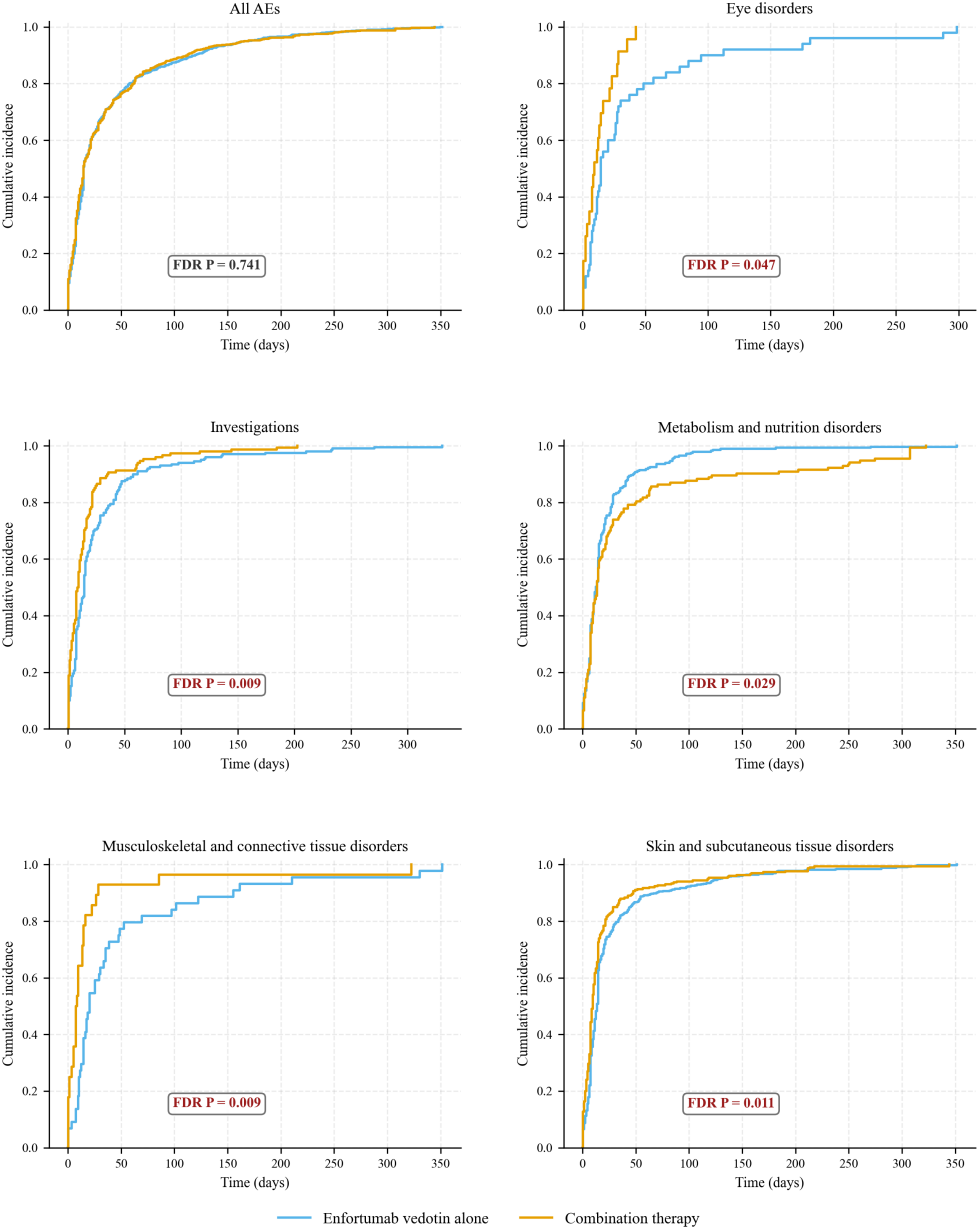
The time to onset for adverse events at the SOC level. The line graph illustrates the trends in cumulative incidence of AEs associated with combination therapy versus monotherapy.

After excluding 3, 241 reports (61.5% of the total cohort) due to missing or illogical date fields, a complete-case analysis was performed on the remaining 2, 028 reports. Overall, the time to onset for all AEs was comparable between the two treatment groups, with an identical median of 14.5 days (FDR P > 0.05). Among the 9 SOCs with disproportionality signals in **Figure 3**, a significant difference in the time to onset was observed only for investigations (FDR P = 0.009), with a median of 14.5 days (IQR: 7.5-28.5 days) for EV monotherapy versus 9 days (IQR: 2.5-17.75 days) for the combination therapy. Additionally, as presented in **Figure 5**, significant differences in the time to onset were also identified for the following SOCs: eye disorders (FDR P = 0.047), metabolism and nutrition disorders (FDR P = 0.029), musculoskeletal and connective tissue disorders (FDR P = 0.009), and skin and subcutaneous tissue disorders (FDR P = 0.011). Except for metabolism and nutrition disorders, where the onset was later with combination therapy, the time to onset for the other 4 SOCs was earlier in the combination group compared to EV monotherapy (**Figure 5**).

## Discussion

Although EV+P has revolutionized UC treatment, a comprehensive real-world safety evaluation remains lacking. Although single-agent safety profiles of EV and pembrolizumab are well-documented, this study provides the first large-scale real-world assessment of the cumulative toxicity risks associated with the EV+P combination. Our findings indicated that the demographic characteristics of patients experiencing AEs were similar between EV monotherapy and combination therapy. AEs mediated by pembrolizumab demonstrated a pronounced risk in the combination group, involving hepatitis, immune-mediated enterocolitis, adrenal insufficiency, hypothyroidism and immune-mediated lung disease. These disproportionality signals perfectly align with the typical irAE profile of pembrolizumab (18). In contrast, typical AEs associated with EV, such as skin toxicity, peripheral neuropathy, hyperglycemia, and ocular disorders, did not show significant exacerbation in the combination group (7). Notably, comparative analysis revealed that combination therapy may elevate the risk of hepatitis and pneumonitis, which was further validated by our systematic literature review. Regarding time to onset, while similar overall between groups, significant variations were noted within 5 SOCs, most of which suggested a potentially earlier onset in the EV + P group.

Furthermore, beyond the previously reported hypertension induced by pembrolizumab, our study identified a distinct cluster of blood pressure–related PTs in the combination group, including blood pressure systolic increased, blood pressure diastolic decreased, and blood pressure fluctuation. Unlike isolated hypertension, this cluster of disproportionality signals suggests a pattern of acute hemodynamic instability and bidirectional blood pressure dysregulation, implying a complex cardiovascular disturbance. Potential mechanisms may involve immune-mediated myocardial injury or autonomic dysfunction associated with pembrolizumab, particularly in the context of myocarditis signals observed in our analysis (6, 9). In addition, EV-related endothelial damage or microtubule disruption induced by MMAE may further exacerbate vascular instability and impair blood pressure regulation (5). However, given the spontaneous reporting nature of FAERS, a definitive causal relationship remains unproven (8).

Our study identified significant hepatotoxicity signals associated with EV + P, evidenced by robust disproportionality signals across the SOC, HLGT, and PT levels. While our observational data cannot confirm biological mechanisms, we hypothesize that the exacerbated hepatic injury might involve a theoretical synergistic interplay between direct cytotoxicity and immune activation. Pembrolizumab was reported to induce immune-related hepatitis characterized by lymphocytic infiltration and lobular hepatitis, occurring in approximately 1.32% of patients (19, 20). Simultaneously, patients receiving EV frequently experience elevated aminotransferases, reflecting direct hepatocellular injury from MMAE metabolism (21). This drug-induced necrosis releases damage-associated molecular patterns (DAMPs), activating innate immunity (22). We speculate that this DAMP-mediated inflammatory milieu could theoretically lower the threshold for immune tolerance, thereby amplifying T-cell-mediated attack on liver tissue and exacerbating the severity of hepatitis. However, it must be emphasized that this proposed mechanism is entirely speculative and requires future experimental validation.

The pulmonary toxicity associated with EV + P is also concerning. Both EV and pembrolizumab can independently induce pneumonitis, which is potentially life-threatening and represents a major cause of treatment discontinuation (23–25). We focused our analysis on pulmonary-related PTs and observed positive signals for pneumonitis, interstitial lung disease, and immune-mediated lung disease, whereas infection-related PTs and malignant/progression-related PTs did not demonstrate significant positive signals. These findings indicate that the respiratory safety profile is more consistent with inflammatory lung toxicity rather than with events driven by infection or malignant processes. Consistently, an integrated analysis of multiple clinical trials demonstrated a higher incidence of pneumonitis in patients treated with EV + P. MMAE-containing ADCs were reported to cause pneumonitis, possibly through non-specific uptake by alveolar macrophages or bystander effects (26, 27). In parallel, PD-1 inhibitors were well documented to cause immune-mediated pneumonitis as a result of T-cell dysregulation within the lung parenchyma (18). On this basis, we hypothesized that MMAE can lead to endothelial or alveolar cell stress/damage in lung tissue via off-target effects, thereby increasing susceptibility to immune-mediated damage. The dual burden of tissue damage plus immune activation may synergistically heighten lung vulnerability (28). A plausible “two-hit” model is therefore that MMAE-related epithelial/endothelial stress and damage-associated molecular pattern release create a local pro-inflammatory substrate, whereas PD-1 blockade lowers immune tolerance and amplifies T-cell-mediated alveolar-interstitial injury. This hypothesis is consistent with our enrichment of inflammatory PTs but cannot be confirmed using FAERS because imaging, microbiology, prior thoracic radiation, baseline interstitial lung disease, and treatment sequence are not consistently available. Nonetheless, given that pneumonitis is frequently associated with severe clinical outcomes, early recognition and timely initiation of corticosteroid therapy are essential.

At the HLGT level, our initial unadjusted analysis flagged hearing disorders and dental/gingival conditions as potential novel risk signals. However, as these signals were based on very small sample sizes with remarkably wide confidence intervals, they failed to maintain statistical significance after FDR correction. We acknowledge that the statistical stability of these specific drug-event pairs is limited, and they carry a high risk of being false positives. While sporadic case reports have linked ICIs to ototoxic events or oral adverse effects (29, 30), the current FAERS data is insufficient to draw definitive conclusions regarding EV+P. Nevertheless, if these ototoxic events are indeed true signals, the underlying mechanism might involve the potent anti-melanocyte activity of pembrolizumab, which damages melanocytes in the inner ear and impairs the maintenance of potassium-rich endolymph within the stria vascularis, ultimately leading to vestibuloauditory dysfunction (31). Overall, these findings must be interpreted strictly as hypothesis-generating, and further validation in larger, prospective cohorts is required before any clinical recommendations can be made.

Several limitations inherent to FAERS data must be acknowledged. First, spontaneous reporting systems carry intrinsic biases, including reporting bias, under-reporting, and duplicate reports. Crucially, the lack of a reliable denominator (total exposed population) precludes calculating true incidence rates or establishing causality. Therefore, the ORs in this study should be interpreted as comparative differences in AE reporting between EV plus pembrolizumab and EV monotherapy, rather than as estimates of true incidence, absolute risk, drug–drug interaction enhancement, or causal effects. Second, reporting rates may be influenced by external factors such as regional reporting practices, differential drug availability, media attention, and stimulated reporting following regulatory updates. Differential reporting between EV+P and EV monotherapy, including heightened clinical awareness of AEs after the approval and clinical use of combination therapy, may inflate disproportionality estimates. Although multivariable adjustment, Primary Suspect-restricted analysis, and ROR shrinkage sensitivity analysis were performed, residual reporting bias and unmeasured confounding cannot be fully excluded. Third, despite a large sample size, FAERS lacks critical clinical details like disease severity, comorbidities, and prior treatments. Additionally, we could not distinguish between concomitant and sequential EV+P administration, potentially introducing misclassification bias. Fourth, time-to-onset estimates require cautious interpretation. Our complete-case analysis excluded 61.5% of reports due to missing or illogical date fields, potentially introducing selection bias. Thus, the observed earlier onset trends for EV+P are exploratory and require prospective validation. Finally, the concentration of EV+P reports from Japan, the United States, and Canada introduces a geographic bias that limits global generalizability. Future research should integrate multiple databases (e.g., JADER, and CVARD) for cross-database validation and pooled analyses to minimize regional bias and enhance the robustness of risk signals.

## Conclusion

This large-scale pharmacovigilance study highlights the distinct and more complex safety profile of the EV+P combination therapy compared to EV monotherapy in the real-world setting. The combination therapy is associated with significantly heightened reporting signals for classic irAEs, particularly endocrine, hepatic, and pulmonary toxicities (e.g., pneumonitis), confirmed by pooled clinical trial incidence data. Potential rare signals, including hearing disorders and dental conditions, warrant further investigation. While the overall median time-to-onset was similar, the potentially earlier onset in specific organ systems with EV+P suggests a need for vigilant early monitoring. These findings bridge the gap between clinical trials and real-world practice, emphasizing the necessity for vigilant, multi-disciplinary monitoring of hepatic, endocrine, and pulmonary systems, especially during early treatment cycles, to promptly manage these complex toxicities and safely optimize this first-line regimen.

## Data Availability

The original contributions presented in the study are included in the article/**Supplementary Material**. Further inquiries can be directed to the corresponding authors.
