# Supplementary material for: The real-world safety profile of enfortumab vedotin with or without pembrolizumab: insights from a comparative analysis of FAERS
Source: Front Immunol. 2026 Jul 24;17:1831172. doi: 10.3389/fimmu.2026.1831172 (PMC13447443; doi:10.3389/fimmu.2026.1831172)
Supplement: Supplementary file 1 [file Table1.docx]

**Table S1.** Focused PT-level regression analysis of pulmonary-related adverse events.

| **Preferred term** | **OR (95% CI)** | ***P* value** |
| --- | --- | --- |
| Pneumonitis | 2.75 (1.61-4.71) | < 0.001 |
| Interstitial lung disease | 3.14 (2.16-4.57) | < 0.001 |
| Immune-mediated lung disease | 4.80 (1.78-12.96) | 0.002 |
| Pneumonia | 1.17 (0.75-1.83) | 0.487 |
| Pneumonia aspiration | 0.85 (0.37-1.97) | 0.708 |
| Metastases to lung | 0.58 (0.29-1.18) | 0.135 |
| Lung disorder | 1.33 (0.55-3.20) | 0.527 |

**Figure S1**. ROR shrinkage sensitivity analysis at the SOC level. sROR was calculated using empirical-Bayes shrinkage of log ROR toward the null value (ROR = 1). Red points indicate positive SOC-level signals that remained robust after shrinkage.

**Figure S2**. Sensitivity analysis restricted to Primary Suspect reports at the SOC level. To assess the influence of drug role coding, the analysis was repeated after restricting the dataset to reports in which the target exposure-defining drug was coded as Primary Suspect. EV monotherapy was used as the reference group. ORs greater than 1 indicate that the corresponding adverse events were more frequently reported in the EV plus pembrolizumab group than in the EV monotherapy group.
